# Heart Failure Independently Predicts Higher Morbidity and Mortality Following Bariatric Surgery: Analysis of 180,544 MBSAQIP Cases

**DOI:** 10.1007/s11695-025-08481-5

**Published:** 2026-01-09

**Authors:** Juan S. Barajas-Gamboa, Valentin Mocanu, Kayanne Khoury, Mélissa V. Wills, Pattharasai Kachornvitaya, Sol Lee, Thomas H. Shin, Matthew Allemang, Andrew T. Strong, Salvador Navarrete, Ricard Corcelles, John Rodriguez, Matthew Kroh, Jerry T. Dang

**Affiliations:** 1 Cleveland Clinic Abu Dhabi, Abu Dhabi, United Arab Emirates; 2 Cleveland Clinic, Cleveland, United States; 3 Royal College of Surgeons in Ireland - Bahrain, Al Muharraq, Bahrain; 4 University of Virginia Health System, Charlottesville, United States; 5 Cleveland Clinic Lerner College of Medicine, Cleveland, United States

**Keywords:** Heart failure, Bariatric surgery, Complications, Gastric bypass, Sleeve gastrectomy, Mortality

## Abstract

**Introduction:**

Heart failure (HF) and obesity represent intersecting public health epidemics with significant healthcare implications. As obesity-related heart failure becomes increasingly prevalent, understanding the perioperative risks and outcomes of bariatric surgery in this vulnerable population is essential. This study aimed to analyze procedure-specific outcomes and cardiac complications in heart failure patients undergoing primary bariatric surgery using a large multi-institutional database.

**Methods and Procedures:**

We conducted a retrospective analysis using the 2023 MBSAQIP database, examining adult patients who underwent primary laparoscopic bariatric procedures (sleeve gastrectomy or Roux-en-Y gastric bypass). Patients were stratified into heart failure and non-heart failure cohorts. The primary outcomes evaluated were 30-day serious complications and mortality. Multivariable logistic regression analyses were performed to identify independent predictors of complications and mortality, adjusting for demographics, comorbidities, and operative factors.

**Results:**

Among 180,544 patients, 2,842 (1.6%) had pre-existing heart failure documented preoperatively. HF patients were significantly older (53.3 ± 11.0 vs. 42.8 ± 11.8 years, *p* < 0.001) with higher BMI (48.6 ± 9.5 vs. 45.0 ± 7.7 kg/m², *p* < 0.001) and greater comorbidity burden. Procedure distribution was similar between groups (SG: 70.7% vs. 72.1%, RYGB: 29.3% vs. 27.9%, *p* = 0.091). HF patients experienced significantly higher rates of serious complications (7.2% vs. 2.4%, *p* < 0.001) and mortality (0.74% vs. 0.06%, *p* < 0.001). Cardiac events occurred 13 times more frequently in HF patients (1.3% vs. 0.1%, *p* < 0.001). Hospital length of stay was longer (1.9 ± 2.9 vs. 1.2 ± 1.0 days, *p* < 0.001), with higher readmission rates (7.8% vs. 2.8%, *p* < 0.001). On multivariable analysis, pre-existing HF was the strongest independent predictor of serious complications (OR 1.81; 95% CI 1.55–2.13; *p* < 0.001) and a powerful predictor of 30-day mortality (OR 3.64; 95% CI 2.14–6.20; *p* < 0.001).

**Conclusion:**

Heart failure is a major independent predictor of both serious complications and mortality following bariatric surgery, occurring in nearly 2% of all elective cases. Despite the established long-term benefits of bariatric surgery for HF patients, the substantially elevated perioperative risks require careful patient selection, thorough preoperative cardiac assessment, and specialized perioperative management strategies to optimize outcomes in this high-risk population.

## Key Points

Pre-existing heart failure is present in 1.6% of bariatric surgery patients and increases postoperative mortality nearly 12-fold.

Heart failure is amongst the strongest independent predictors of serious complications (OR 1.81).

Heart failure independently predicts 30-day mortality (OR 3.64) after bariatric surgery.

Cardiac events are 13 times more frequent in heart failure patients undergoing bariatric surgery.

Heart failure patients experience longer hospital stays and higher readmission rates.

## Introduction

Obesity and heart failure (HF) represent two intersecting public health epidemics with significant implications for healthcare systems worldwide. The prevalence of both conditions continues to rise, with over 5.7 million adults in the United States suffering from heart failure—a number projected to increase by 46% by 2030 [1]. This trend is particularly concerning given heart failure’s substantial mortality burden, with reported 30-day, 1-year, and 5-year mortality rates of 10%, 20–30%, and over 75%, respectively [2, 3].

Obesity is a well-established independent risk factor for HF development and progression [4–6]. Approximately 30–49% of HF patients have concomitant obesity [7], creating a complex clinical scenario that necessitates optimized management strategies addressing both conditions simultaneously.

Bariatric surgery has emerged as the most effective treatment for severe obesity, providing substantial and sustained weight loss along with significant improvements in obesity-related comorbidities [8]. For patients with HF specifically, weight loss through bariatric surgery offers several potential benefits, including improved cardiac function, reduced ventricular filling pressures, enhanced exercise capacity, and decreased HF exacerbations requiring hospitalization [9–11]. These hemodynamic and symptomatic improvements can translate to meaningful gains in quality of life and potentially reduced long-term morbidity.

Despite these established benefits, the perioperative management of HF patients undergoing bariatric surgery presents unique challenges. These patients inherently carry a higher surgical risk profile due to compromised cardiac function, reduced hemodynamic reserve, and an elevated burden of comorbidities [12]. The most commonly performed bariatric procedures—Roux-en-Y gastric bypass (RYGB) and sleeve gastrectomy (SG)—while generally safe and effective for weight loss [13–15], may present different risk-benefit profiles in this vulnerable population.

The optimal approach to procedure selection, perioperative management, and risk stratification for HF patients undergoing bariatric surgery remains incompletely defined. While several studies have examined outcomes of bariatric surgery in patients with obesity-related HF [8, 9, 12, 16, 17], most are limited by small sample sizes, single-centre experiences, or lack of procedure-specific analyses. Large-scale, multi-institutional data on this specific patient population are lacking, creating a critical knowledge gap for clinicians managing these complex patients.

The Metabolic and Bariatric Surgery Accreditation and Quality Improvement Program (MBSAQIP) database provides a unique opportunity to examine nationwide outcomes in this population with robust statistical power. This study analyzes procedure-specific 30-day outcomes, mortality, and cardiac complications in HF patients undergoing primary bariatric surgery, while examining prevalence, short-term outcomes, and predictors of serious complications and mortality. This analysis aims to provide clinicians with evidence-based guidance for procedure selection and risk stratification when considering bariatric surgery for patients with concurrent heart failure.

## Materials and Methods

### Study Design and Data Source

This retrospective cohort study analyzed prospectively collected data from the (MBSAQIP database for the year 2023. The MBSAQIP is a nationally validated registry containing detailed clinical data from 902 accredited bariatric surgery centres across the United States and Canada. Dedicated clinical reviewers at each participating institution collect data on patient demographics, preoperative comorbidities, operative details, and 30-day postoperative outcomes using standardized definitions. It is important to note that 2023 is the only year which captures HF data. Data quality is ensured through regular auditing processes and training of clinical reviewers.

### Ethical Considerations

This study was reviewed by the Institutional Review Board and deemed exempt from the requirement for formal approval due to the de-identified nature of the data. The MBSAQIP participant use file contains no protected health information and complies with the Health Insurance Portability and Accountability Act (HIPAA) regulations for use in research.

### Study Population and Inclusion Criteria

The study cohort included adult patients (≥ 18 years) who underwent primary laparoscopic bariatric procedures in 2023. Patients were identified using Current Procedural Terminology (CPT) codes for laparoscopic sleeve gastrectomy (43775) and laparoscopic Roux-en-Y gastric bypass (43644). Only primary (first-time) bariatric procedures were included in the analysis. Patients with HF were identified using the specific variable “hrt_fail” in the MBSAQIP database. All variable definitions and standardized data collection methods are detailed in the 2023 MBSAQIP Participant Use Data File User Guide, which is publicly available through the American College of Surgeons. The study population was divided into two cohorts: (1) patients with pre-existing HF and (2) patients without heart failure. Patients with missing data on HF status or procedure type were excluded from the analysis.

### Study Objectives

The primary objectives of this study were to analyze procedure-specific 30-day outcomes, mortality, and cardiac complications in HF patients undergoing primary bariatric surgery, and to identify independent predictors of serious complications and 30-day mortality.

### Variables and Outcomes

#### Patient Characteristics

Demographic variables included age, sex, race/ethnicity, and body mass index (BMI). Preoperative comorbidities were extracted from the MBSAQIP database, including hypertension, diabetes (non-insulin and insulin-dependent), obstructive sleep apnea, chronic obstructive pulmonary disease (COPD), hyperlipidemia, gastroesophageal reflux disease (GERD), renal insufficiency, history of venous thromboembolism (VTE), history of myocardial infarction (MI), and therapeutic anticoagulation use. Preoperative risk factors included American Society of Anesthesiologists (ASA) Physical Status classification and functional status (independent, partially dependent, or totally dependent).

#### Operative Variables

The primary operative variables included procedure type (sleeve gastrectomy or Roux-en-Y gastric bypass) and operative time. All procedures were performed using a laparoscopic and robotic approach.

#### Outcome Measures

The primary outcome was the occurrence of serious complications and mortality within 30 days of surgery. A serious complication was defined as a composite outcome including one or more of the following: bleeding requiring transfusion, deep surgical site infection, anastomotic leak, pneumonia, pulmonary embolism, acute kidney injury, myocardial infarction, cardiac arrest requiring cardiopulmonary resuscitation, unplanned intubation, sepsis, venous thromboembolism, cerebrovascular accident, reoperation, or reintervention.

Secondary outcomes included individual components of the serious complications composite, hospital readmission within 30 days, length of hospital stay, and procedure-specific complications. Cardiac-specific complications were analyzed separately, including myocardial infarction, cardiac arrest, and other cardiac events as defined in the MBSAQIP database.

### Statistical Analysis

Descriptive statistics were used to summarize baseline characteristics and outcomes. Categorical variables were expressed as frequencies and percentages, while continuous variables were reported as means with standard deviations. Comparisons between HF and non-heart failure patients were performed using chi-square tests for categorical variables and independent-sample t-tests for continuous variables.

Univariate logistic regression was initially performed to identify potential risk factors for serious complications. Variables with a p-value < 0.05 in univariate analysis were included in the multivariable logistic regression model to identify independent predictors of serious complications. The multivariable model adjusted for the following covariates: age, sex, BMI, diabetes, history of myocardial infarction, operative time, hypertension, procedure type (bypass vs. sleeve), renal insufficiency, functional status, COPD, history of VTE, race, and therapeutic anticoagulation. A separate multivariable logistic regression model was developed to identify independent predictors of 30-day mortality, adjusting for the same covariates as in the serious complications model.

Variables were checked for collinearity using the variance inflation factor method, with a threshold of > 5 indicating significant collinearity. The predictive performance of the multivariable model was assessed using the area under the receiver operating characteristic curve (AUROC) and Brier score for calibration. The available case method was used to address missing data, as all variables had less than 5% missingness. Results are presented as adjusted odds ratios (OR) with 95% confidence intervals (CI). A two-sided p-value < 0.05 was considered statistically significant. All statistical analyses were performed using STATA version 17 statistical software (StataCorp, College Station, TX, USA).

## Results

### Study Population Characteristics

Among 180,544 patients who underwent primary laparoscopic bariatric surgery in 2023, 2,842 (1.6%) had a documented history of HF. Patients with HF were significantly older than those without HF (53.3 ± 11.0 vs. 42.8 ± 11.8 years, *p* < 0.001) and had a higher mean BMI (48.6 ± 9.5 vs. 45.0 ± 7.7 kg/m², *p* < 0.001). HF patients were more likely to be male (38.0% vs. 17.5%, *p* < 0.001) and had a higher proportion of Black patients (28.5% vs. 19.9%, *p* < 0.001) compared to those without HF (Table 1)**.**

**Table 1:** Demographics and preoperative characteristics of patients undergoing primary bariatric surgery

Characteristic	Heart Failure Patients (*n* = 2,842)	Non-Heart Failure Patients (*n* = 177,702)	*P*-value
Demographics			
Age, mean ± SD (years)	53.3 ± 11.0	42.8 ± 11.8	< 0.001
Female sex, n (%)	1,763 (62.0)	146,557 (82.5)	< 0.001
BMI, mean ± SD (kg/m²)	48.6 ± 9.5	45.0 ± 7.7	< 0.001
Race, n (%)			< 0.001
White	1,705 (60.0)	109,256 (61.5)	
Black	811 (28.5)	35,418 (19.9)	
Other	326 (11.5)	33,028 (18.6)	
Preoperative Characteristics			
ASA Classification, n (%)			< 0.001
ASA Class I-II	65 (2.3)	31,613 (17.8)	
ASA Class III	2,185 (76.9)	141,113 (79.5)	
ASA Class IV-V	591 (20.8)	4,827 (2.7)	
Functional Status, n (%)			< 0.001
Independent	2,717 (95.8)	176,739 (99.5)	
Partially dependent	114 (4.0)	801 (0.5)	
Totally dependent	6 (0.2)	33 (< 0.1)	
Comorbidities, n (%)			
Hypertension	2,550 (89.7)	76,222 (42.9)	< 0.001
Diabetes	1,489 (52.4)	41,123 (23.1)	< 0.001
- Insulin-dependent	648 (22.8)	9,927 (5.6)	< 0.001
Sleep apnea	2,076 (73.1)	66,741 (37.6)	< 0.001
COPD	358 (12.6)	1,805 (1.0)	< 0.001
Hyperlipidemia	1,740 (61.2)	40,621 (22.9)	< 0.001
GERD	1,214 (42.7)	52,573 (29.6)	< 0.001
Renal insufficiency	161 (5.7)	836 (0.5)	< 0.001
History of VTE	346 (12.2)	4,607 (2.6)	< 0.001
History of MI	356 (12.5)	1,460 (0.8)	< 0.001
Therapeutic anticoagulation	793 (27.9)	4,661 (2.6)	< 0.001
Current smoker	215 (7.6)	10,883 (6.1)	0.002

Abbreviations: *SD* Standard Deviation, *BMI* Body Mass Index, *ASA* American Society of Anesthesiologists, *COPD* Chronic Obstructive Pulmonary Disease, *GERD* Gastroesophageal Reflux Disease, *VTE* Venous Thromboembolism, *MI* Myocardial Infarction

HF patients had significantly higher rates of preoperative comorbidities, including hypertension (89.7% vs. 42.9%, *p* < 0.001), diabetes (52.4% vs. 23.1%, *p* < 0.001), sleep apnea (73.1% vs. 37.6%, *p* < 0.001), COPD (12.6% vs. 1.0%, *p* < 0.001), hyperlipidemia (61.2% vs. 22.9%, *p* < 0.001), and renal insufficiency (5.7% vs. 0.5%, *p* < 0.001). Notably, HF patients had substantially higher rates of previous myocardial infarction (12.5% vs. 0.8%, *p* < 0.001) and venous thromboembolism (12.2% vs. 2.6%, *p* < 0.001), with more frequent use of therapeutic anticoagulation (27.9% vs. 2.6%, *p* < 0.001) **(**Table 1**).**

HF patients also demonstrated higher preoperative surgical risk, with a greater proportion classified as ASA class IV-V (20.8% vs. 2.7%, *p* < 0.001) and more patients with partially or totally dependent functional status (4.2% vs. 0.5%, *p* < 0.001).

### Operative Characteristics

The distribution of primary bariatric procedures was similar between HF and non-HF patients, with no statistically significant difference in the rates of sleeve gastrectomy (70.7% vs. 72.1%, *p* = 0.091) and Roux-en-Y gastric bypass (29.3% vs. 27.9%, *p* = 0.091). However, HF patients had significantly longer operative times (98.6 ± 56.1 vs. 84.4 ± 48.7 min, *p* < 0.001) **(**Table 2**).**

**Table 2:** Operative details and 30-Day outcomes of primary bariatric surgery

Variable	Heart Failure Patients (*n* = 2,842)	Non-Heart Failure Patients (*n* = 177,702)	*P*-value
Operative Details			
Sleeve gastrectomy, n (%)	2,008 (70.7)	128,103 (72.1)	0.091
Roux-en-Y gastric bypass, n (%)	834 (29.3)	49,599 (27.9)	0.091
Operative time, mean ± SD (min)	98.6 ± 56.1	84.4 ± 48.7	< 0.001
30-day Outcomes			
Mortality, n (%)	21 (0.74)	107 (0.06)	< 0.001
Serious complications, n (%)	205 (7.2)	4,316 (2.4)	< 0.001
Cardiac events, n (%)	37 (1.3)	158 (0.1)	< 0.001
Bleeding, n (%)	75 (2.6)	1,422 (0.8)	< 0.001
Deep surgical site infection, n (%)	18 (0.6)	452 (0.3)	< 0.001
Pneumonia, n (%)	22 (0.7)	295 (0.1)	< 0.001
Pulmonary embolism, n (%)	11 (0.4)	188 (0.1)	< 0.001
Venous thromboembolism, n (%)	23 (0.8)	589 (0.3)	< 0.001
Acute kidney injury, n (%)	25 (0.9)	158 (0.09)	< 0.001
Myocardial infarction, n (%)	10 (0.3)	48 (0.03)	< 0.001
Unplanned intubation, n (%)	23 (0.8)	135 (0.1)	< 0.001
Sepsis, n (%)	10 (0.4)	120 (0.1)	< 0.001
Gastrointestinal leak, n (%)	14 (0.5)	331 (0.2)	< 0.001
Wound disruption, n (%)	4 (0.1)	58 (0.03)	0.002
Bowel obstruction, n (%)	20 (0.7)	560 (0.3)	< 0.001
Hospital readmission, n (%)	221 (7.8)	4,996 (2.8)	< 0.001
Reoperation, n (%)	46 (1.6)	1,436 (0.8)	< 0.001
Reintervention, n (%)	53 (1.9)	1,087 (0.6)	< 0.001
Length of stay, mean ± SD (days)	1.9 ± 2.9	1.2 ± 1.0	< 0.001

Abbreviations: *SD* Standard Deviation, *min* minutes

### 30-day Complications Associated with HF Outcomes Using Bivariate Analysis

HF patients experienced substantially worse 30-day outcomes compared to patients without heart failure. Mortality was markedly higher in HF patients (0.74% vs. 0.06%, *p* < 0.001), as was the overall rate of serious complications (7.2% vs. 2.4%, *p* < 0.001) **(**Table 2**).**

Specific complications that occurred at significantly higher rates in HF patients included cardiac events (1.3% vs. 0.1%, *p* < 0.001), bleeding (2.6% vs. 0.8%, *p* < 0.001), acute kidney injury (0.4% vs. 0.04%, *p* < 0.001), myocardial infarction (0.3% vs. 0.03%, *p* < 0.001), and unplanned intubation (0.8% vs. 0.1%, *p* < 0.001). Infectious complications were also more common in HF patients, including pneumonia (0.7% vs. 0.1%, *p* < 0.001), deep surgical site infections (0.6% vs. 0.3%, *p* < 0.001), and sepsis (0.4% vs. 0.1%, *p* < 0.001) **(**Table 2**).**

Thromboembolic complications showed similar patterns, with HF patients having higher rates of pulmonary embolism (0.4% vs. 0.1%, *p* < 0.001) and overall venous thromboembolism (0.8% vs. 0.3%, *p* < 0.001). Gastrointestinal complications such as anastomotic leaks (0.5% vs. 0.2%, *p* < 0.001) and bowel obstruction (0.7% vs. 0.3%, *p* < 0.001) were also more frequent in the HF cohort **(**Table 2**).**

HF patients required more unplanned interventions, with higher rates of hospital readmission (7.8% vs. 2.8%, *p* < 0.001), reoperation (1.6% vs. 0.8%, *p* < 0.001), and reintervention (1.9% vs. 0.6%, *p* < 0.001). Additionally, HF patients had a longer mean length of hospital stay (1.9 ± 2.9 vs. 1.2 ± 1.0 days, *p* < 0.001) **(**Table 2**).**

### Multivariable Analysis for Serious Complications

After adjusting for demographics, comorbidities, and operative factors in multivariable logistic regression analysis, HF remained one of the strongest independent predictors of serious complications within 30 days of bariatric surgery (adjusted OR 1.81; 95% CI 1.55–2.13; *p* < 0.001) **(**Table 3**).**

**Table 3:** Multivariable logistic regression analysis of predictors of 30-Day serious complications following primary bariatric surgery

Variable	Adjusted Odds Ratio	95% CI	*P*-value
Heart failure	1.81	1.55–2.13	< 0.001
Age (per 10-year increase)	1.07	1.04–1.11	< 0.001
Female sex	0.89	0.83–0.96	0.004
BMI (per 5-unit increase)	1.01	0.99–1.03	0.401
Diabetes			
- Non-insulin dependent	1.01	0.93–1.09	0.836
- Insulin dependent	1.16	1.04–1.29	0.009
History of myocardial infarction	1.33	1.08–1.65	0.007
Operative time (per minute)	1.003	1.003–1.004	< 0.001
Hypertension	1.18	1.10–1.26	< 0.001
Roux-en-Y gastric bypass	1.73	1.61–1.85	< 0.001
Renal insufficiency	1.32	0.99–1.75	0.057
Partially dependent functional status	1.63	1.23–2.16	0.001
COPD	1.57	1.31–1.90	< 0.001
History of VTE	1.50	1.30–1.74	< 0.001
Therapeutic anticoagulation	1.40	1.22–1.61	< 0.001
Race			
- Black (vs. White)	1.20	1.11–1.29	< 0.001
- Other (vs. White)	0.95	0.87–1.03	0.233

Abbreviations: *CI* Confidence Interval, *BMI* Body Mass Index, *COPD* Chronic Obstructive Pulmonary Disease, *VTE* Venous Thromboembolism

*Note: Model adjusted for age, sex, body mass index, comorbidities, and operative time. n=179,449. Area under the receiver operating characteristic curve = 0.65, Brier score = 0.024

Other significant independent predictors of serious complications included increased age (adjusted OR 1.07 per 10-year increase; 95% CI 1.04–1.11; *p* < 0.001), insulin-dependent diabetes (adjusted OR 1.16; 95% CI 1.04–1.29; *p* = 0.009), history of myocardial infarction (adjusted OR 1.33; 95% CI 1.08–1.65; *p* = 0.007), increased operative time (adjusted OR 1.003 per minute; 95% CI 1.003–1.004; *p* < 0.001), hypertension (adjusted OR 1.18; 95% CI 1.10–1.26; *p* < 0.001), Roux-en-Y gastric bypass procedure (adjusted OR 1.73; 95% CI 1.61–1.85; *p* < 0.001), partially dependent functional status (adjusted OR 1.63; 95% CI 1.23–2.16; *p* = 0.001), COPD (adjusted OR 1.57; 95% CI 1.31–1.90; *p* < 0.001), history of VTE (adjusted OR 1.50; 95% CI 1.30–1.74; *p* < 0.001), therapeutic anticoagulation (adjusted OR 1.40; 95% CI 1.22–1.61; *p* < 0.001), and Black race (adjusted OR 1.20 vs. White; 95% CI 1.11–1.29; *p* < 0.001) **(**Table 3**).**

Female sex was associated with lower odds of serious complications (adjusted OR 0.89; 95% CI 0.83–0.96; *p* = 0.004). BMI did not emerge as an independent predictor of serious complications in the multivariable model (adjusted OR 1.01 per 5-unit increase; 95% CI 0.99–1.03; *p* = 0.401) **(**Table 3**).**

### Multivariable Analysis for Mortality

In the multivariable logistic regression model for mortality, HF demonstrated a powerful independent association with 30-day mortality following bariatric surgery when controlling for patient demographics, comorbidities, and operative characteristics (adjusted OR 3.64; 95% CI 2.14–6.20; *p* < 0.001) **(**Table 4**).**

**Table 4:** Multivariable logistic regression analysis of predictors of 30-Day mortality following primary bariatric surgery

Variable	Adjusted Odds Ratio	95% CI	*P*-value
Heart failure	3.64	2.14–6.20	< 0.001
Age (per 10-year increase)	1.69	1.42–2.01	< 0.001
Female sex	0.70	0.48–1.03	0.073
BMI (per 5-unit increase)	1.42	1.31–1.55	< 0.001
Diabetes			
- Non-insulin dependent	1.07	0.69–1.65	0.767
- Insulin dependent	1.96	1.21–3.16	0.006
History of myocardial infarction	0.18	0.03–1.33	0.094
Operative time (per minute)	1.005	1.002–1.007	0.001
Hypertension	1.79	1.12–2.85	0.015
Roux-en-Y gastric bypass	1.21	0.81–1.80	0.344
Renal insufficiency	1.02	0.31–3.33	0.975
Partially dependent functional status	0.88	0.27–2.88	0.833
COPD	1.11	0.50–2.49	0.795
History of VTE	1.63	0.85–3.12	0.141
Therapeutic anticoagulation	1.25	0.68–2.29	0.474
Race			
- Black (vs. White)	1.40	0.93–2.10	0.109
- Other (vs. White)	0.94	0.53–1.67	0.834

Abbreviations: *CI * Confidence Interval, *BMI* Body Mass Index, *COPD* Chronic Obstructive Pulmonary Disease, *VTE* Venous Thromboembolism

*Note: Model adjusted for age, sex, body mass index, comorbidities, and operative time. n=179,449 with 128 mortality events (0.07% crude mortality rate). Area under the receiver operating characteristic curve = 0.84, Brier score = 0.024

Other significant independent predictors of mortality included increased age (adjusted OR 1.69; 95% CI 1.42–2.01; *p* < 0.001), higher BMI (adjusted OR 1.42; 95% CI 1.31–1.55; *p* < 0.001), insulin-dependent diabetes (adjusted OR 1.96; 95% CI 1.21–3.16; *p* = 0.006), and increased operative time (adjusted OR 1.005 per minute; 95% CI 1.002–1.007; *p* = 0.001). Hypertension was also associated with higher mortality risk (adjusted OR 1.79; 95% CI 1.12–2.85; *p* = 0.015) **(**Table 4**)**.

Female sex showed a trend toward lower mortality risk, though this did not reach statistical significance (adjusted OR 0.70; 95% CI 0.48–1.03; *p* = 0.073). Notably, history of myocardial infarction was not significantly associated with mortality in the multivariable model (adjusted OR 0.18; 95% CI 0.03–1.33; *p* = 0.094) **(**Table 4**)**.

## Discussion

This study represents the largest analysis to date examining bariatric surgery outcomes specifically in patients with heart failure, encompassing 2,842 HF patients among 180,544 primary procedures from the 2023 MBSAQIP database. Our principal finding is that pre-existing heart failure is among the strongest independent predictors of both serious complications (adjusted OR 1.81) and 30-day mortality (adjusted OR 3.64) following bariatric surgery, with HF patients experiencing 12-fold higher crude mortality rates compared to those without HF. These elevated risks extend across multiple complication domains, including cardiac, infectious, thromboembolic, and renal events, underscoring the vulnerability of this population during the perioperative period.

While patients with HF carry a substantially higher baseline surgical risk due to their impaired cardiac function and associated comorbidities, several studies have highlighted the benefits of performing bariatric surgery in patients with obesity and with established heart failure, including significant weight loss, improvement in cardiac function, a decrease in the burden of comorbidities and metabolic derangements, and ultimately a meaningful gain in quality of life [16]. Studies have also evaluated the role of bariatric surgery as a bridge to cardiac transplantation and placement of a left ventricular assist device (LVAD), allowing patients with severe obesity – who were previously disqualified due to their BMI – to be considered for these life-saving therapies [9, 12, 18]. Though some previous studies found bariatric surgery to be relatively safe in patients with HF with no increase in short-term mortality, these benefits must be carefully weighed against the significantly increased risks we identified [19, 20], our data demonstrated HF as being the greatest predictor of mortality, with a nearly 4-fold increase in mortality rates within 30 days of primary bariatric surgery.

Our findings diverge from some previous literature in important ways. For instance, although Vest et al. also found that HF patients had a greater risk of myocardial infarction 30 days postoperatively (2% vs. 0.04%, *p* = 0.032), there was no statistically significant difference in the occurrence of venous thromboembolism, AKI, pneumonia, bleeding, sepsis, or wound infection [20]. In contrast, our much larger sample size revealed statistically significant increases in all these complications, suggesting previous studies may have been underpowered to detect these differences. Moreover, patients with HF in our study had a mean length of stay of 1.9 days ± 2.9, whereas that of HF patients in studies by Strong et al. [21] and Yang et al. [12] reported a mean length of stay of 3.57 days ± 3.15 and 3.6 days ± 4.2, respectively. This notable difference may also be attributed to the significant difference in sample populations. This difference may also reflect the general trend toward shorter postoperative stays and same-day discharge protocols in bariatric surgery that have evolved since those earlier studies from 2018 to 2020. However, the significantly higher complication rates observed in HF patients in our study suggest that current same-day discharge protocols may need modification for this population, potentially incorporating risk stratification tools and tailored monitoring approaches.

Reoperation rates were also significantly greater (1.6%) in the HF cohort. This was consistent with another study which demonstrated that patients with HF had a significantly greater chance (5.9% vs. 2.3%, *p* < 0.005) of returning to the operating theatre within 30 days of primary bariatric surgery [21]. A limitation of the MBSAQIP database is that it does not provide specific indications for these reoperations. HF patients likely experience higher reoperation rates due to compromised tissue perfusion, which can increase risks of anastomotic leaks and wound complications that require surgical intervention. The underlying hemodynamic compromise and potential fluid shifts in these patients may impair healing processes factors that should be considered in perioperative management but require more detailed clinical data to fully evaluate [2, 3].

Our analysis also revealed a significant difference in the rates of readmission between both cohorts, with 7.8% of HF patients being readmitted within 30 days compared to 2.8% in non-heart failure patients. This nearly three-fold higher readmission rate suggests that HF patients may benefit from extended inpatient monitoring before discharge and more intensive post-discharge follow-up protocols. Early discharge decisions that might be appropriate for standard bariatric patients could be premature for those with heart failure, who may develop complications more gradually or after initial discharge. More robust transition-of-care programs with earlier post-discharge follow-up visits, remote monitoring of vital signs and symptoms, and heart failure-specific discharge education might help reduce these readmission rates. Other studies, however, found no significant difference in readmission rates [12, 20]. The preoperative severity of HF and reason for readmission – both of which were unknown to us in our study – are vital in order to appropriately draw conclusions that explain these findings.

The strengths of our study include the utilization of a large-scale and multi-institutional database with comprehensive national representation. The MBSAQIP registry provides standardized data collection across hundreds of accredited centers, maximizing the generalizability of our results. The large sample size allowed us to detect clinically significant differences that might not be apparent in smaller studies. Our identification of HF as a major independent predictor of both complications and mortality provides a valuable guide for clinical practice. These findings support the implementation of specialized care pathways for bariatric patients with heart failure, including mandatory preoperative cardiology consultation, comprehensive echocardiographic assessment, optimization of medical therapy, management of associated conditions such as atrial fibrillation, and tailored perioperative anticoagulation protocols [6, 7]. At our institutions, we have implemented a structured approach involving preoperative HF optimization, stratification based on ventricular function and pulmonary pressures, and selective use of advanced hemodynamic monitoring during surgery for high-risk cases. This multidisciplinary approach that includes cardiac specialists in the perioperative team may help mitigate the substantially elevated risks we identified while preserving access to the metabolic benefits of bariatric surgery for this vulnerable population.

However, important limitations must be acknowledged. First, our study analyzed data collected solely from the 2023 MBSAQIP database, which is a limited time frame. Moreover, the data provided is limited to 30 days postoperatively and thus limits our ability to assess the long-term postoperative complications. Second, and most critically, the MBSAQIP database lacks granularity regarding heart failure severity and phenotype - limitations with profound implications for interpreting our findings. Heart failure is a heterogeneous syndrome encompassing distinct phenotypes with markedly different pathophysiology and perioperative risk profiles. Heart failure with reduced ejection fraction (HFrEF, LVEF < 40%) and heart failure with preserved ejection fraction (HFpEF, LVEF ≥ 50%) - both highly prevalent in obesity - likely carry different perioperative risks. HFrEF is characterized by systolic dysfunction with reduced contractile reserve, while HFpEF features diastolic dysfunction with stiff ventricles highly sensitive to volume status. Perioperative management strategies and risk profiles likely differ substantially between these phenotypes.

Without information on left ventricular ejection fraction, NYHA functional classification, HF etiology, compensation status, or medical optimization, we cannot stratify risk within the heart failure population or determine whether our associations apply equally across this heterogeneous spectrum. For example, well-compensated HFpEF on optimal therapy may face substantially different risks than severely reduced LVEF with recent decompensation. Similarly, patients on advanced HF therapies or being bridged to transplantation represent distinct subpopulations with unique risk-benefit considerations not captured in our aggregate analysis.

This limitation is particularly important when considering procedure selection. While our multivariable analysis demonstrates that both heart failure (OR 1.81) and RYGB (OR 1.73) independently predict serious complications, we did not perform formal interaction testing or procedure-stratified analyses to determine whether heart failure differentially modifies the risk associated with RYGB compared to sleeve gastrectomy. The similar distribution of sleeve gastrectomy and RYGB between HF and non-HF cohorts (70.7% vs. 29.3% compared to 72.1% vs. 27.9%, *p* = 0.091) may reflect contemporary practice patterns where sleeve gastrectomy has become the predominant procedure overall, or it may obscure important variations in disease severity that influenced procedure selection. It is plausible that patients with more advanced or poorly compensated HF were preferentially offered sleeve gastrectomy due to shorter operative times and reduced physiologic stress, while those with better-compensated HF may have been considered acceptable candidates for RYGB, particularly when metabolic comorbidities favored bypass.

The inability to stratify by HF severity, ejection fraction, or phenotype fundamentally limits our ability to provide phenotype-specific guidance. The absence of interaction analyses limit our ability to determine whether the effects of HF and procedure type are purely additive or whether there is synergistic risk. Third, our multivariable model for serious complications demonstrated modest discrimination with an AUROC of 0.65, indicating that while we identified several significant independent predictors, substantial variability in complication risk remains unexplained by the variables available in the MBSAQIP database. This modest discriminatory ability likely reflects the multifactorial and complex nature of postoperative complications, which are influenced not only by patient demographics and comorbidities but also by unmeasured factors such as specific heart failure phenotypes (HFrEF vs. HFpEF), cardiac functional capacity (NYHA class, ejection fraction), perioperative optimization strategies, intraoperative hemodynamic management, surgeon experience, institutional protocols, and other clinical nuances not captured in administrative databases.

In contrast, our mortality prediction model demonstrated better discrimination (AUROC = 0.84), suggesting that prediction of this more severe and definitive outcome is more robust with the available clinical variables. However, the relatively low number of mortality events (*n* = 128) in our analysis, while reflecting the generally low mortality of contemporary bariatric surgery, results in approximately 7 events per variable in our 18-variable mortality model. This approaches the lower limit of traditional modeling guidelines and may affect model stability and the precision of individual coefficient estimates, particularly for variables with low prevalence such as history of myocardial infarction.

Indeed, the counterintuitive point estimate for history of MI in the mortality model (OR 0.18, 95% CI 0.03–1.33, *p* = 0.094) exemplifies this instability. This finding, which would implausibly suggest a protective effect of prior MI, almost certainly reflects collinearity between MI and heart failure (given that 12.5% of HF patients had prior MI compared to only 0.8% of non-HF patients) combined with the small number of mortality events. The wide confidence interval crossing 1.0 and non-significant p-value appropriately indicate the uncertainty around this estimate. While we included history of MI a priori based on its established clinical importance as a cardiovascular risk factor, this finding underscores the limitations of multivariable modeling with relatively few outcome events and highlights the need for cautious interpretation of individual coefficient estimates beyond our primary predictor of interest (HF).

Despite this limitation, the overall model performance (AUROC = 0.84) and the consistency of our primary findings with existing literature support the validity of our conclusions regarding heart failure as a major independent predictor of mortality. The difference in model performance highlights that while mortality risk can be reasonably stratified using conventional risk factors, the prediction of non-fatal complications requires more granular clinical data than is available in large registry databases. Future studies incorporating detailed cardiac functional assessments, procedure-stratified outcome analyses, and formal interaction testing would be valuable in guiding procedure selection for this heterogeneous patient population based on individual cardiac risk profiles.

Additionally, we lack nuanced information on operative technique, surgeon case load, method of perioperative optimization, post-operative monitoring protocols, and other variables which may confound our results. Furthermore, we are unable to identify which patients had left ventricular assist devices (LVADs) or were undergoing bariatric surgery as a bridge to cardiac transplantation, information that would be valuable for risk stratification and outcomes analysis in this specialized population.

Third, while we identified HF as the strongest predictor of complications, and a major independent predictor of mortality, we cannot establish causality from this observational study. Selection bias may also influence our results, as patients with severe HF may be less likely to be offered bariatric surgery, potentially underestimating the true risk in the full spectrum of HF patients.

Future research should focus on prospective studies with extended follow-up to better assess the long-term implications of bariatric surgery in patients with heart failure. Development of heart failure-specific risk calculators for bariatric surgery candidates would greatly enhance clinical decision-making. Additionally, studies investigating optimal perioperative management strategies specifically tailored for HF patients undergoing bariatric surgery are needed to potentially mitigate some of the risks we identified.

## Conclusion

This study provides compelling evidence that HF is the strongest independent predictor of serious complications and mortality following primary bariatric surgery. HF patients demonstrated an 81% higher risk of serious complications and a 12-fold higher crude mortality rate (0.74% vs. 0.06%), with HF increasing the odds of death more than 3.6-fold even after adjustment for numerous confounders. However, these findings represent aggregate risks across a heterogeneous HF population and cannot distinguish between different phenotypes (HFrEF vs. HFpEF), severity levels (NYHA Class I-IV), or compensation status - factors that profoundly influence perioperative risk. Therefore, our results should not be applied uniformly to all patients with heart failure, and individual risk assessment with detailed cardiac phenotyping remains essential.

Although the long-term benefits of bariatric surgery for HF patients are well-established in the literature, our findings highlight the critical importance of meticulous perioperative optimization and risk stratification in this vulnerable population. Bariatric surgery in HF patients requires careful patient selection, thorough preoperative cardiac assessment, and specialized perioperative management to minimize the substantially increased risk of adverse outcomes we observed. Future research with granular cardiac functional data is essential to develop phenotype-specific guidelines for this high-risk population.

## Data Availability

No datasets were generated or analysed during the current study.

## Abbreviations

SD: Standard Deviation
BMI: Body Mass Index
ASA: American Society of Anesthesiologists
COPD: Chronic Obstructive Pulmonary Disease
GERD: Gastroesophageal Reflux Disease
VTE: Venous Thromboembolism
MI: Myocardial Infarction
